# The Role of Vimentin, Synaptophysin, and Histone H3 Lysine 27 Methylation (H3K27me) as Surrogate Markers in the Diagnosis and Classification of Oligodendrogliomas and Diffuse Midline Gliomas: A Comprehensive Review

**DOI:** 10.7759/cureus.89806

**Published:** 2025-08-11

**Authors:** Hussein Qasim, Karees Khattab, Mohammad Abu Shugaer, Giustino Varrassi

**Affiliations:** 1 Department of Pathology and Laboratory Medicine, Jordan University of Science and Technology, Irbid, JOR; 2 Faculty of Medicine, Jordan University of Science and Technology, Irbid, JOR; 3 Department of Pain Medicine, Fondazione Paolo Procacci, Rome, ITA

**Keywords:** diffuse midline glioma, glioma, h3k27me3, immunohistochemistry staining, oligodendroglioma, synaptophysin, vimentin

## Abstract

Oligodendrogliomas and diffuse midline gliomas (DMGs) are distinct subtypes of central nervous system (CNS) tumors with differing prognoses and treatment responses. Accurate differentiation between these tumors is critical yet often challenging, particularly when comprehensive molecular testing is unavailable. This review explores the diagnostic value of immunohistochemical surrogate markers, vimentin, synaptophysin, and histone H3 lysine 27 methylation (H3K27me), in distinguishing these tumor types. A narrative review methodology was employed following the Scale for the Assessment of Narrative Review Articles (SANRA) guidelines. Relevant peer-reviewed studies were identified through comprehensive database searches of PubMed, Embase, Scopus, Web of Science (WoS), and Google Scholar using targeted keywords and Medical Subject Headings (MeSH) terms. Articles were included based on their focus on the immunohistochemical and molecular characterization of oligodendrogliomas and DMGs. Vimentin, typically associated with mesenchymal transition, is highly expressed in DMGs and high-grade gliomas, reflecting aggressive behavior and invasiveness. In contrast, oligodendrogliomas usually lack vimentin expression. Synaptophysin, a neuronal differentiation marker, is frequently expressed in oligodendrogliomas but largely absent in DMGs, offering discriminatory value. The loss of H3K27 trimethylation (H3K27me3) expression is a defining feature of H3K27M-mutant DMGs and serves as a highly specific diagnostic marker. Together, these markers improve diagnostic precision, particularly in resource-limited settings or where molecular assays are not readily available. Vimentin, synaptophysin, and H3K27me expression patterns offer practical, cost-effective surrogate tools to enhance diagnostic accuracy in glioma classification. Their integration into routine neuropathological assessment can support timely and appropriate therapeutic decision-making, especially in settings lacking full molecular testing capabilities. Further research is needed to explore their potential roles in guiding targeted therapies and prognostication.

## Introduction and background

Gliomas are the most common primary tumors of the central nervous system (CNS), comprising a diverse group of neoplasms with distinct histopathological and molecular characteristics [1]. Among them, oligodendrogliomas and diffuse midline gliomas (DMGs) represent two important subtypes that require accurate differentiation due to their prognostic and therapeutic implications [2]. Advances in neuro-oncology have led to the integration of molecular markers into the World Health Organization (WHO) Classification of CNS Tumors, refining glioma diagnosis beyond conventional histopathology [3]. However, distinguishing these entities remains challenging, particularly in cases where histological features overlap or when comprehensive molecular testing is unavailable [4]. This challenge is further exacerbated in low-resource settings, where access to advanced molecular diagnostics such as next-generation sequencing (NGS) or fluorescence in situ hybridization (FISH) is limited by cost, infrastructure, and expertise. In such contexts, reliable surrogate immunohistochemical markers become critical for accurate classification and treatment guidance.

Oligodendrogliomas are characterized by isocitrate dehydrogenase (IDH) mutation and 1p/19q co-deletion, a molecular signature that defines their diagnosis [4]. These tumors typically exhibit a well-differentiated morphology, round nuclei with perinuclear halos (fried-egg appearance), and a delicate capillary network [5]. In contrast, diffuse midline gliomas, particularly the H3K27M-mutant subgroup, represent a distinct and highly aggressive class of gliomas that arise in midline structures such as the thalamus, pons, and spinal cord [6]. DMGs are diffusely infiltrative tumors with poor prognosis, often resistant to conventional therapy [2]. Given these challenges, immunohistochemistry (IHC) remains a crucial tool for supporting glioma diagnosis in routine clinical practice [7]. In particular, vimentin, synaptophysin, and histone H3 lysine 27 methylation (H3K27me) have gained attention as potential surrogate markers that aid in differentiating oligodendroglioma from DMGs [8-10]. Vimentin, a mesenchymal cytoskeletal protein, is upregulated in more aggressive gliomas and is typically expressed in DMGs, reflecting their high invasiveness [10]. In contrast, oligodendrogliomas usually exhibit low vimentin expression, which can aid in their distinction [11]. Synaptophysin, a neuronal differentiation marker, is often expressed in oligodendrogliomas due to their partial neuronal-like differentiation [12].

DMGs, on the other hand, tend to lack strong synaptophysin positivity, making this marker a potential differentiating factor [13]. H3K27me, referring to histone H3 lysine 27 methylation, plays a critical role in epigenetic regulation [14]. The loss of H3K27 trimethylation (H3K27me3) staining is a hallmark of H3K27M-mutant DMGs, making this marker particularly valuable for identifying this highly aggressive glioma subtype [15]. The use of these surrogate markers in diagnostic pathology enhances accuracy, particularly in cases where molecular testing is unavailable, inconclusive, or cost-prohibitive [16]. Moreover, their expression patterns provide insight into tumor biology, prognosis, and potential therapeutic strategies [17]. The 2021 World Health Organization (WHO) Classification of CNS Tumors redefined glioma taxonomy by integrating molecular criteria into traditional histological assessment [3]. This update emphasized the importance of IDH mutation status, 1p/19q co-deletion, and H3K27 alterations in stratifying gliomas into biologically and clinically distinct subtypes. These changes are especially significant for pathologists and clinicians in refining diagnoses and therapeutic approaches [3]. This review explores the diagnostic significance of vimentin, synaptophysin, and H3K27me, highlighting their role in glioma classification and their potential utility in differentiating oligodendrogliomas from DMGs. By examining their expression patterns, clinical relevance, and limitations, this discussion aims to provide a comprehensive perspective on their use in neuropathology and neuro-oncology practice.

## Review

Methodology

This narrative review utilizes a descriptive thematic approach to examine the diagnostic and prognostic utility of vimentin, synaptophysin, and H3K27me as surrogate immunohistochemical markers in differentiating oligodendrogliomas from diffuse midline gliomas (DMGs). The review is structured in accordance with the Scale for the Assessment of Narrative Review Articles (SANRA) guidelines to ensure quality, transparency, and methodological rigor in the review process. Eligible literature included peer-reviewed articles focused on the diagnostic, histopathological, immunohistochemical, or molecular characterization of oligodendrogliomas and DMGs, with a specific emphasis on vimentin, synaptophysin, or H3K27me expression. Accepted study types included original research articles, systematic reviews, meta-analyses, case series, and expert commentaries. Publications were required to be available in full-text English. Studies were excluded if they lacked relevance to the diagnostic utility of the specified markers, did not provide interpretive data or methodological clarity, or were not peer-reviewed. A comprehensive literature search was performed across the following databases: PubMed, Scopus, Embase, Web of Science (WoS), and Google Scholar. Search terms were structured using Medical Subject Headings (MeSH) and Boolean operators to maximize sensitivity and specificity. The primary search string used was: ("vimentin" OR "synaptophysin" OR "H3K27me3" OR "H3K27M") AND ("oligodendroglioma" OR "diffuse midline glioma" OR "glioma classification" OR "brain tumor diagnosis"). Searches were further refined by screening reference lists of selected papers to capture relevant studies not retrieved in the initial database queries.

WHO classification and molecular landscape

The 2021 WHO Classification of CNS Tumors emphasizes the use of molecular features to classify gliomas into more biologically and clinically relevant subgroups [18]. Gliomas are now primarily divided based on their genetic alterations rather than histological appearance alone [19]. Oligodendrogliomas are defined as IDH-mutant and 1p/19q-co-deleted gliomas [19]. These genetic alterations distinguish oligodendrogliomas from diffuse astrocytomas, which lack the 1p/19q co-deletion and often harbor ATRX mutations [20]. The co-deletion of chromosomes 1p and 19q results from an unbalanced whole-arm translocation between these chromosomes and is strongly associated with improved response to therapy and prolonged survival [21]. In contrast, the defining molecular feature of these tumors is the H3K27M mutation, which involves a lysine-to-methionine substitution at position 27 of histone H3 genes (*H3F3A*, *HIST1H3B*, or *HIST1H3C*) [22]. This mutation disrupts histone methylation, leading to the global loss of H3K27 trimethylation (H3K27me3), which in turn silences tumor suppressor genes and promotes an aggressive tumor phenotype [23].

Oligodendrogliomas tend to be less aggressive, have better response to treatment, and exhibit longer survival rates, whereas DMGs are highly infiltrative, resistant to conventional therapy, and associated with poor prognosis [24]. Histologically, oligodendrogliomas exhibit a "fried-egg" appearance, characterized by round nuclei surrounded by clear perinuclear halos [25]. The tumor vasculature often forms a delicate, branching "chicken-wire" pattern, and calcifications are frequently present [26]. Immunohistochemically, IDH1 R132H mutation is commonly detected using IHC staining, though sequencing may be required for rare IDH mutations [27]. ATRX protein expression is retained, distinguishing oligodendrogliomas from astrocytomas, which frequently lose ATRX expression [28]. Olig2, a marker of oligodendroglial lineage, is consistently positive, and synaptophysin expression may be seen in some cases, reflecting neuronal differentiation [29]. 1p/19q co-deletion detection is performed using fluorescence in situ hybridization (FISH) or polymerase chain reaction (PCR) [30]. IDH mutation confirmation is also necessary, particularly if IHC results are inconclusive [31]. These molecular markers help distinguish oligodendrogliomas from diffuse astrocytomas, which lack 1p/19q co-deletion and often harbor TP53 mutations [20].

DMGs are histologically characterized by diffuse, infiltrative growth involving midline structures of the brain and spinal cord [32]. High-grade features such as nuclear atypia, mitotic activity, necrosis, and microvascular proliferation are commonly observed [33]. The presence of pseudopalisading necrosis is a hallmark of more aggressive cases [34]. IHC staining is essential for diagnosing DMGs as the loss of H3K27me3 expression is a key marker of H3K27M-mutant gliomas and is widely used to confirm the diagnosis [34]. Glial fibrillary acidic protein (GFAP) positivity indicates astrocytic differentiation, while high Ki-67 (proliferation index) values reflect tumor aggressiveness [35]. In addition, vimentin positivity is often observed, particularly in tumors exhibiting mesenchymal-like transformation [36]. Molecular testing plays a definitive role in confirming DMG diagnosis [37]. H3K27M mutation detection is performed using next-generation sequencing (NGS) or PCR [22]. Additional alterations, such as TP53 mutations and activin A receptor type 1 (ACVR1) mutations, are frequently found in DMGs and may provide further diagnostic insight [38]. MGMT promoter methylation testing is also performed to assess the tumor's response to alkylating chemotherapy agents such as temozolomide [39]. A comparative summary of the histopathological, molecular, and clinical characteristics distinguishing oligodendrogliomas from diffuse midline gliomas is presented in Table 1.

**Table 1:** Comparison of key clinical, histopathological, molecular, and immunohistochemical features of oligodendrogliomas and diffuse midline gliomas (DMGs). WHO, World Health Organization; IDH, isocitrate dehydrogenase; GFAP, glial fibrillary acidic protein; PCV, procarbazine, lomustine, and vincristine; H3K27me3, histone H3 lysine 27 trimethylation

Feature	Oligodendroglioma	Diffuse Midline Glioma (DMG)
WHO grade	Grade 2 or 3 [40]	Grade 4 [41]
Cell of origin	Oligodendrocytes [42]	Astrocytes (glial cells) [43]
Histopathology	"Fried-egg" cells, chicken-wire vasculature, and calcifications [20]	Diffuse infiltration, necrosis, and microvascular proliferation [2]
Key molecular alterations	IDH1/2 mutation and 1p/19q co-deletion [44]	H3K27M mutation and H3K27me3 loss [45]
Immunohistochemistry	ATRX retained, Olig2+, and synaptophysin+ (variable) [27]	GFAP+, vimentin+, Ki-67 high, and H3K27me3 loss [46]
Prognosis	Better prognosis with standard therapy [47]	Poor prognosis and median survival of <1 year [2]
Therapy response	Responsive to radiotherapy and chemotherapy (PCV and temozolomide) [48]	Poor response to standard therapy and clinical trials for targeted treatments [2]

Vimentin as a diagnostic marker

Vimentin is a type 3 intermediate filament protein that plays a fundamental role in maintaining cell structure, adhesion, and migration [49]. It is primarily expressed in mesenchymal-derived cells, including fibroblasts, endothelial cells, and immune cells [49]. In tumor pathology, vimentin is widely recognized as a marker of epithelial-to-mesenchymal transition (EMT), a process associated with tumor progression, increased invasiveness, and therapy resistance [36]. Given its role in tumor biology, vimentin has gained significant attention as a diagnostic and prognostic marker in gliomas [50]. In neuro-oncology, vimentin expression is often correlated with tumor aggressiveness, particularly in high-grade gliomas [50]. While it is not entirely specific to any single glioma subtype, its differential expression patterns across various gliomas make it a useful surrogate marker in tumor classification [51]. Low-grade gliomas and well-differentiated tumors typically exhibit weak or absent vimentin staining, whereas highly invasive and mesenchymal-like gliomas show strong vimentin positivity [52]. This distinction highlights its diagnostic utility in distinguishing oligodendrogliomas from diffuse midline gliomas (DMGs) and astrocytomas [53].

Vimentin plays a crucial role in tumor progression by influencing cellular plasticity, invasion, and survival under stress conditions [36]. Its function in gliomas can be summarized by three key mechanisms. First, vimentin enhances tumor invasion and migration, enabling glioma cells to infiltrate the surrounding brain tissue [54]. High vimentin expression is often associated with more aggressive gliomas, where tumor cells exhibit increased motility and capacity to invade healthy neural structures [55]. This property is particularly evident in diffuse gliomas, which lack well-defined borders and spread extensively within the brain [55]. Second, vimentin contributes to epithelial-to-mesenchymal transition (EMT), a process linked to therapy resistance [56]. EMT allows glioma cells to adopt a mesenchymal phenotype, making them more resistant to chemotherapy and radiotherapy [57]. This is particularly relevant in glioblastomas (GBMs), which are known for their treatment resistance and frequent recurrence [58]. Third, vimentin plays a role in angiogenesis and the tumor microenvironment [59]. Its expression in tumor-associated endothelial cells promotes the formation of new blood vessels, supporting tumor growth and survival [59]. Additionally, glioma stem-like cells (GSCs), which contribute to tumor recurrence and therapy resistance, have been shown to express high levels of vimentin, further implicating its role in glioma pathogenesis [60]. The level of vimentin expression varies across different glioma subtypes, providing important insights into their biological behavior and diagnostic classification [8].

Oligodendrogliomas typically exhibit low or absent vimentin expression, reflecting their well-differentiated nature and epithelial-like morphology [61]. These tumors, defined by IDH mutations and 1p/19q co-deletion, tend to be less infiltrative than astrocytomas and high-grade gliomas [62]. The lack of vimentin expression in oligodendrogliomas helps distinguish them from more aggressive gliomas that show mesenchymal features [8]. In contrast, DMGs, particularly those with the H3K27M mutation, demonstrate moderate to high vimentin expression [63]. These tumors are highly infiltrative and exhibit significant therapy resistance, characteristics that align with the increased vimentin positivity observed in their tumor cells [64]. The presence of vimentin in DMGs correlates with their poor prognosis and rapid progression [64]. The expression pattern in astrocytomas (IDH-mutant, WHO grades 2-4) is more variable [65]. While low-grade astrocytomas (grades 2 and 3) may show focal or weak vimentin staining, high-grade astrocytomas and glioblastomas (GBM, WHO grade 4) frequently exhibit strong and diffuse vimentin expression [66]. The transition from lower- to higher-grade astrocytomas is often accompanied by an increase in vimentin expression, further supporting its role as a marker of tumor progression [64]. Other CNS tumors, such as ependymomas, meningiomas, and choroid plexus tumors, may also express vimentin, though its diagnostic significance in these tumors is limited compared to glioma [67]. Given its differential expression across glioma subtypes, vimentin serves as an important surrogate marker in tumor classification, particularly when molecular testing is unavailable or inconclusive [8].

Oligodendrogliomas typically lack vimentin expression, while DMGs exhibit moderate to strong vimentin positivity [11]. This distinction is particularly useful in cases where H3K27M mutation testing is not readily available, as vimentin expression can support the identification of highly aggressive DMGs [15]. Vimentin also plays a role in distinguishing low-grade gliomas from glioblastomas [54]. Low-grade astrocytomas and oligodendrogliomas tend to exhibit weak or absent vimentin staining, whereas glioblastomas consistently show strong and widespread vimentin positivity [52]. This expression pattern correlates with the mesenchymal transformation observed in glioblastomas, which is associated with their highly invasive nature and therapy resistance [68]. Furthermore, vimentin can be used alongside other markers to improve diagnostic accuracy [64]. For example, combining vimentin with H3K27me3 staining can help confirm H3K27M-mutant DMGs, which show both high vimentin expression and the loss of H3K27me3 staining [69]. In contrast, oligodendrogliomas typically retain H3K27me3 expression and lack vimentin positivity [70]. As shown in Table 2, vimentin expression is low or absent in oligodendrogliomas, whereas it is strong and diffuse in glioblastomas and high-grade astrocytomas, highlighting its value in distinguishing tumor aggressiveness. Vimentin serves as a valuable surrogate marker for aggressive glioma phenotypes, particularly DMGs. Its absence in oligodendrogliomas and high expression in mesenchymal-transformed gliomas support its role in differentiating glioma subtypes when molecular testing is unavailable.

**Table 2:** Vimentin expression patterns across glioma subtypes and their diagnostic significance. WHO, World Health Organization; IDH, isocitrate dehydrogenase; CNS, central nervous system

Glioma Type	Vimentin Expression	Diagnostic Significance
Oligodendroglioma (IDH-mutant and 1p/19q-co-deleted) [71]	Low/negative	Helps distinguish between astrocytic gliomas and diffuse midline gliomas (DMGs)
Diffuse midline glioma (H3K27M-mutant, WHO grade 4) [72]	Moderate to high	Reflects aggressive behavior and helps differentiate from oligodendroglioma
Astrocytomas (IDH-mutant, WHO grades 2-4) [73]	Variable	Focal in lower grades and stronger in high-grade astrocytomas
Glioblastoma (IDH-wildtype, WHO grade 4) [74]	Strong and diffuse	Correlates with mesenchymal transition and poor prognosis
Meningioma and choroid plexus tumors [67]	Positive	Not specific to gliomas but seen in other CNS tumors

Synaptophysin: Expression patterns and diagnostic utility

Synaptophysin is a presynaptic vesicle glycoprotein involved in synaptic transmission and neuroendocrine differentiation [75]. It is widely expressed in neuronal and neuroendocrine tissues and is commonly used as an immunohistochemical (IHC) marker to assess neuronal differentiation in central nervous system (CNS) tumors [76]. In gliomas, synaptophysin expression varies significantly across subtypes, reflecting differences in their lineage differentiation and molecular characteristics [77]. Among gliomas, oligodendrogliomas frequently express synaptophysin, indicating a degree of neuronal differentiation, while DMGs typically lack strong synaptophysin positivity [78]. This differential expression makes synaptophysin a useful surrogate marker for distinguishing oligodendrogliomas from other gliomas, particularly astrocytic tumors such as DMGs [20]. However, synaptophysin is not entirely specific to oligodendrogliomas and should always be interpreted in conjunction with other molecular and histological markers [20]. Synaptophysin is a crucial synaptic vesicle protein that facilitates neurotransmitter release at synapses [79]. It is predominantly expressed in neuronal and neuroendocrine cells, making it an essential marker for identifying tumors with neuronal differentiation [80]. The presence of synaptophysin in gliomas suggests a degree of neuronal lineage differentiation, which is more commonly observed in oligodendrogliomas than in astrocytic gliomas [81]. This differentiation pattern is thought to arise due to the cellular origins of oligodendrogliomas, which display features of both glial and neuronal lineage.

Beyond gliomas, synaptophysin is also expressed in neuroendocrine tumors, pheochromocytomas, paragangliomas, medulloblastomas, and central neurocytomas [82]. In these contexts, synaptophysin is used to differentiate neuronal and neuroendocrine tumors from purely glial tumors, highlighting its significance in CNS tumor classification [83]. The expression pattern of synaptophysin differs significantly between oligodendrogliomas and diffuse midline gliomas, making it a useful diagnostic tool in neuropathology [84]. Oligodendrogliomas frequently exhibit synaptophysin positivity, supporting the notion that these tumors retain neuronal-like differentiation [20]. Immunohistochemically, synaptophysin staining in oligodendrogliomas is often focal or patchy, with cytoplasmic and perinuclear positivity [81]. The degree of synaptophysin expression can vary, but its presence supports the diagnosis of oligodendroglioma over astrocytic tumors [85]. In summary, synaptophysin is a useful immunohistochemical marker for identifying neuronal differentiation, with strong expression typically seen in oligodendrogliomas and absent or weak expression in DMGs. While not specific to gliomas alone, its presence supports an oligodendroglial phenotype, especially when interpreted alongside molecular markers. Despite some variability, synaptophysin remains a practical diagnostic tool in differentiating glioma subtypes when used as a part of a multi-marker panel.

Synaptophysin expression in diffuse midline gliomas (DMGs)

In contrast, diffuse midline gliomas (DMGs) typically lack significant synaptophysin expression [2]. These tumors, particularly those with the H3K27M mutation, are predominantly astrocytic in origin, showing little to no evidence of neuronal differentiation [86]. Most DMGs exhibit absent or weak synaptophysin staining, a feature that helps differentiate them from oligodendrogliomas [12]. In some cases, scattered synaptophysin positivity may be detected, but it is not a defining characteristic of DMGs [87]. Instead, DMGs strongly express glial fibrillary acidic protein (GFAP) and vimentin, markers associated with astrocytic and mesenchymal differentiation rather than neuronal differentiation [88]. Given these differences, strong synaptophysin positivity favors an oligodendroglioma diagnosis, while negative or weak synaptophysin staining in a midline tumor supports a diagnosis of DMG [89]. While synaptophysin is a valuable marker in glioma classification, it has limitations that must be considered when interpreting its expression.

Diagnostic specificity

Synaptophysin is not exclusive to oligodendrogliomas and is expressed in various other CNS tumors, including neurocytomas, which exhibit strong synaptophysin positivity [90]; gangliogliomas, which contain both glial and neuronal components [91]; and dysembryoplastic neuroepithelial tumors (DNETs) [92], which also demonstrate neuronal differentiation, in addition to certain astrocytic tumors, which may show focal synaptophysin expression [93]. Because synaptophysin is expressed in multiple tumor types, its positivity alone cannot confirm an oligodendroglioma diagnosis [94]. It must be interpreted alongside molecular markers such as IDH mutation, 1p/19q co-deletion, ATRX, and H3K27me3 for a definitive classification [95]. Potential limitations of synaptophysin include variable expression in oligodendrogliomas because while most oligodendrogliomas are synaptophysin-positive, some may exhibit weak or absent staining [20]. This variability can lead to false-negative results, particularly in small biopsy samples. Another limitation is the focal versus diffuse staining patterns as in oligodendrogliomas, synaptophysin expression is often focal rather than diffuse [96]. Small biopsy samples may not capture representative staining, leading to diagnostic uncertainty. Moreover, synaptophysin has a limited utility in high-grade gliomas; for example, high-grade gliomas (glioblastomas and anaplastic astrocytomas) often lose synaptophysin expression as they become more undifferentiated [97], reducing its reliability as a prognostic marker in aggressive gliomas [98]. Despite these limitations, synaptophysin remains a valuable marker when used alongside other diagnostic tools. Its presence strongly supports oligodendroglioma, while its absence favors DMGs or high-grade astrocytic gliomas.

H3K27me3 loss in diffuse midline gliomas: Epigenetic marker and clinical implications

Histone modifications play a crucial role in the epigenetic regulation of gene expression, influencing chromatin structure and cellular differentiation [99]. One of the most well-studied histone modifications in gliomas is H3K27 methylation (H3K27me), which involves the methylation of lysine 27 on histone H3 [14]. This modification is essential for transcriptional repression and gene silencing, particularly in the regulation of developmental genes [14]. In diffuse midline gliomas (DMGs), a defining molecular alteration is the H3K27M mutation, which disrupts normal histone methylation patterns [100]. This mutation is highly specific to DMGs and serves as a critical diagnostic and prognostic biomarker [100]. The loss of H3K27 trimethylation (H3K27me3) due to this mutation profoundly affects gene expression, contributing to gliomagenesis and tumor progression [101]. Histone H3 lysine 27 methylation (H3K27me) is a key epigenetic modification regulated by the polycomb repressive complex 2 (PRC2) [102], which includes enhancer of zeste homolog 2 (EZH2), suppressor of zeste 12 homolog (SUZ12), and embryonic ectoderm development (EED) proteins [103].

PRC2 catalyzes the trimethylation of H3K27 (H3K27me3), leading to the silencing of genes involved in cell differentiation and proliferation [104]. This regulatory mechanism is important for normal neural development and the maintenance of cellular identity. In H3K27M-mutant DMGs, a missense mutation in the *H3F3A*, *HIST1H3B*, or *HIST1H3C* genes results in the substitution of lysine (K) with methionine (M) at position 27 of histone H3 [22]. This mutation inhibits PRC2 activity, preventing the normal trimethylation of H3K27 [22]. As a result, genes that are normally silenced by PRC2 become aberrantly activated, leading to uncontrolled cell proliferation, the loss of differentiation, and enhanced tumor aggressiveness [105]. The disruption of transcriptional repression allows oncogenic pathways to remain active, contributing to the highly infiltrative and treatment-resistant nature of these tumors [106]. The impact of the H3K27M mutation extends beyond gene activation [107]. It creates a widespread epigenetic reprogramming in tumor cells, leading to alterations in chromatin architecture and transcriptional landscapes [108]. These reprogramming drives tumor heterogeneity, allowing cells to evade differentiation signals and resist conventional treatments such as chemotherapy and radiotherapy [109]. Due to these mechanisms, H3K27M-mutant DMGs are highly aggressive tumors with a poor prognosis, often leading to patient mortality within one year of diagnosis [110].

The identification of H3K27M mutation has revolutionized the classification of diffuse midline gliomas, distinguishing them from other gliomas based on their unique molecular and histopathological features [15]. Unlike other gliomas, DMGs exhibit a highly infiltrative growth pattern and arise predominantly in midline structures, including the thalamus, pons, and spinal cord [2]. Their aggressive nature and poor response to therapy make them one of the most challenging CNS tumors to treat. Histopathologically, H3K27M-mutant DMGs display diffuse infiltration, high nuclear atypia, increased mitotic activity, and frequent necrosis with microvascular proliferation [2]. These tumors often show astrocytic morphology, although some may appear undifferentiated or primitive. Immunohistochemically, H3K27me3 loss is the hallmark feature of these tumors, distinguishing them from other gliomas that retain normal H3K27 methylation [10]. GFAP and vimentin are often expressed in DMGs, reinforcing their astrocytic lineage, while the Ki-67 proliferation index is typically high, indicating rapid tumor growth [88]. Molecularly, H3K27M-mutant DMGs lack 1p/19q co-deletion, differentiating them from oligodendrogliomas, and they are also IDH-wildtype, which helps distinguish them from lower-grade astrocytomas [111]. Unlike glioblastomas, which can arise in any brain region, DMGs are confined to the midline structures, making the H3K27M mutation a highly specific biomarker for their diagnosis [100]. Given its specificity, H3K27M mutation testing is now a critical step in glioma classification and helps guide clinical decision-making [100]. In summary, the loss of H3K27me3 expression is a hallmark of H3K27M-mutant DMGs and serves as a highly specific and clinically relevant diagnostic marker. This epigenetic alteration underlies the aggressive behavior of these tumors and is critical for accurate classification. As molecular testing expands, H3K27me3 IHC continues to provide a reliable surrogate for identifying this biologically distinct and therapeutically challenging glioma subtype.

Challenges and future perspectives

Despite advancements in understanding H3K27M mutation in DMGs, several challenges remain in their diagnosis and treatment [112]. One of the biggest diagnostic challenges is the heterogeneity of histone modifications within tumors [113]. While most tumor cells in H3K27M-mutant DMGs lose H3K27me3 expression, some regions may retain partial methylation, leading to the potential misinterpretation of IHC results [114]. Additionally, small biopsy samples may not be representative of the entire tumor, making molecular confirmation via sequencing essential in ambiguous cases [115]. From a therapeutic perspective, H3K27M-mutant DMGs remain highly resistant to conventional treatments, including radiotherapy and chemotherapy [116]. The lack of effective targeted therapies is a major challenge, and efforts are underway to develop novel epigenetic inhibitors that can restore normal histone methylation patterns [117]. Among the emerging therapeutic strategies, EZH2 inhibitors aim to restore PRC2 function, while histone deacetylase (HDAC) inhibitors modulate chromatin structure to counteract epigenetic dysregulation [118]. Bromodomain and extraterminal (BET) protein inhibitors are also being explored for their role in targeting transcriptional regulators that promote tumor growth [119]. Immunotherapy is another area of active research, with ongoing trials investigating checkpoint inhibitors and chimeric antigen receptor T (CAR-T) cell therapies for DMGs [120]. However, the success of these approaches has been limited due to the immunosuppressive tumor microenvironment of DMGs [121]. Overcoming this barrier will require a deeper understanding of the tumor's immune landscape and the development of strategies to enhance immune response against H3K27M-mutant cells.

Clinical and diagnostic implications

The accurate diagnosis of gliomas is crucial for determining prognosis and guiding therapeutic strategies [122]. The integration of immunohistochemical (IHC) and molecular markers, such as vimentin, synaptophysin, and H3K27me, has significantly enhanced the ability to distinguish between different glioma subtypes, particularly oligodendrogliomas and diffuse midline gliomas (DMGs) [15,70]. These markers not only improve diagnostic accuracy but also provide insights into tumor behavior, prognosis, and potential treatment responses [123]. However, while the use of these markers in combination strengthens diagnostic confidence, certain limitations and gaps in research must still be addressed to optimize their clinical utility [123]. Beyond diagnosis, these markers have significant implications for prognosis and treatment strategies [124]. Gliomas exhibit widely variable clinical outcomes, and molecular markers have become essential in stratifying patients based on expected tumor behavior and response to therapy [125]. H3K27M-mutant DMGs are highly aggressive tumors with poor prognosis, typically having a median survival of less than one year despite treatment [126]. The loss of H3K27me3 expression is strongly correlated with high tumor proliferation, therapy resistance, and rapid disease progression [127]. Identifying this marker in a patient's tumor provides important prognostic information, enabling clinicians to discuss expectations with patients and prioritize enrollment in clinical trials investigating novel therapeutic agents, such as EZH2 inhibitors, histone deacetylase (HDAC) inhibitors, and immunotherapy approaches [128].

**Figure 1:**
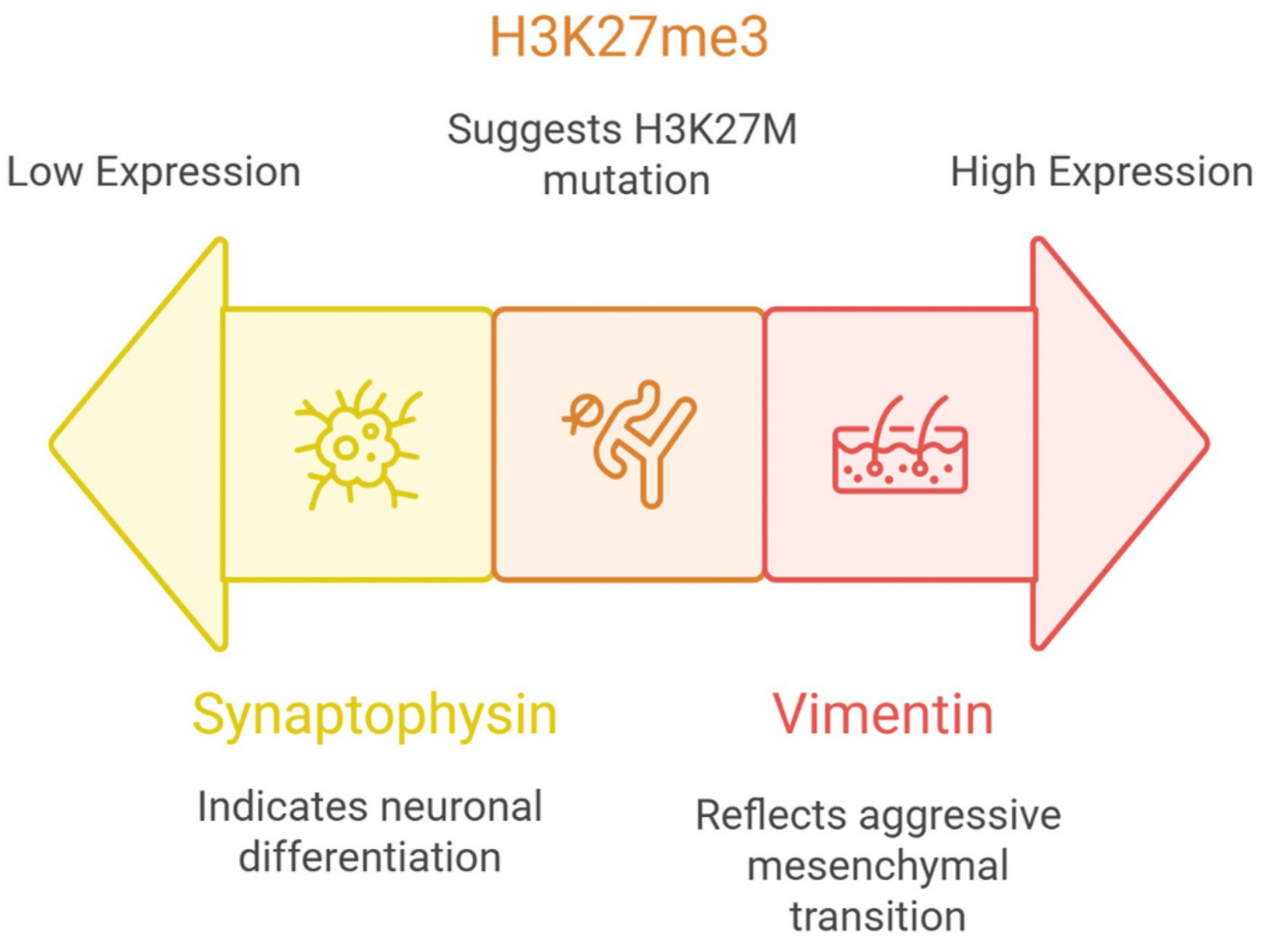
Visual representation of glioma tumor types distinguished by immunohistochemical marker expression levels. The figure was created by the team and is original and not taken from any external resource. Image credits: Karees Khattab. H3K27me3: histone H3 lysine 27 trimethylation

Oligodendrogliomas, on the other hand, tend to have a better prognosis, particularly those with IDH mutation and 1p/19q co-deletion [129]. The presence of synaptophysin positivity supports an oligodendroglioma diagnosis and is often associated with a more indolent course and better response to chemotherapy and radiotherapy [130]. These tumors generally respond well to standard treatments, such as procarbazine, lomustine, and vincristine (PCV) chemotherapy, as well as temozolomide-based regimens [131]. Vimentin expression, when present in gliomas, is often associated with a more aggressive, therapy-resistant phenotype [132]. In high-grade gliomas, strong vimentin expression correlates with epithelial-to-mesenchymal transition (EMT), increased tumor invasiveness, and poor treatment response [133]. This suggests that vimentin-positive gliomas may require more aggressive treatment strategies, and future research is exploring whether vimentin expression could serve as a predictive biomarker for targeted therapies aimed at inhibiting mesenchymal transition in gliomas [134]. As research into glioma biology advances, these markers may also play a role in guiding personalized treatment approaches [135]. For example, tumors with high vimentin expression may be more responsive to therapies targeting tumor invasion and migration, while synaptophysin-positive gliomas could benefit from differentiation-inducing therapies [64]. H3K27M-mutant gliomas, given their epigenetic dysregulation, are an important target for novel histone-modifying enzyme inhibitors currently in development [136,137]. The relative expression patterns of synaptophysin, H3K27me3, and vimentin across glioma subtypes are summarized visually in Figure 1.

Limitations and gaps in current research

Despite the promising clinical applications of vimentin, synaptophysin, and H3K27me, there remain several limitations and gaps in research that must be addressed to further optimize their use in glioma diagnostics and treatment planning. One major limitation is heterogeneity in marker expression within gliomas [138]. Some gliomas may exhibit mixed expression patterns, leading to challenges in interpretation [139]. For example, while H3K27M-mutant DMGs are typically negative for synaptophysin, rare cases may show focal positivity, making it difficult to rely on a single marker for diagnosis [2]. Similarly, vimentin expression can be variable within gliomas, particularly in those undergoing histological transformation to higher-grade tumors [50]. This heterogeneity emphasizes the need for multi-marker panels and molecular confirmation whenever possible. Another limitation is the potential for false-negative or false-positive results in IHC staining [140]. Small biopsy samples may not capture representative tumor regions, leading to sampling bias [141]. Additionally, technical variability in IHC protocols across laboratories can affect staining intensity and interpretation [142]. For example, H3K27me3 staining may appear reduced rather than completely absent in some DMGs, making the distinction from other gliomas more challenging. The standardization of IHC protocols and the expanded use of molecular validation techniques could help mitigate these issues.

Furthermore, while these markers provide valuable prognostic information, they have not yet been fully integrated into treatment decision-making frameworks [143]. The role of vimentin in guiding therapy selection remains largely unexplored, and more research is needed to determine whether synaptophysin-positive gliomas have unique therapeutic vulnerabilities [144]. Additionally, while epigenetic therapies targeting H3K27M-mutant DMGs are under investigation, their clinical efficacy remains uncertain, and more studies are needed to establish effective treatment regimens [145]. Finally, access to molecular and IHC testing remains a barrier in some clinical settings, particularly in low-resource environments. While the use of surrogate markers such as vimentin and synaptophysin can aid in glioma classification when molecular testing is unavailable, efforts should be made to improve global access to genetic testing technologies, ensuring that all patients receive accurate diagnoses and appropriate treatment recommendations.

## Conclusions

The accurate classification and diagnosis of gliomas, particularly oligodendrogliomas and diffuse midline gliomas (DMGs), are critical for guiding prognosis and treatment. In the absence of comprehensive molecular testing, immunohistochemical markers such as vimentin, synaptophysin, and H3K27me serve as valuable surrogate tools to differentiate between these glioma subtypes. Vimentin's association with aggressive, mesenchymal-like transformation supports its utility in identifying high-grade gliomas such as DMGs, while synaptophysin expression points toward neuronal differentiation, favoring a diagnosis of oligodendroglioma. The loss of H3K27me3 staining remains a hallmark of H3K27M-mutant DMGs and provides key diagnostic specificity. Although these markers significantly enhance diagnostic accuracy and offer insight into tumor biology, limitations such as intratumoral heterogeneity, sampling variability, and technical differences in staining protocols highlight the need for careful interpretation. As our molecular understanding of gliomas evolves, integrating IHC markers with genetic testing will remain central to improving diagnostic precision, prognostic stratification, and, ultimately, personalized therapeutic strategies. Future research should focus on standardizing diagnostic approaches, expanding access to molecular diagnostics, and exploring the predictive potential of these markers in therapeutic response.
